# Structural and dynamical properties of water adsorption on PtO_2_(001)

**DOI:** 10.1039/c8ra00952j

**Published:** 2018-04-20

**Authors:** Yong Yang

**Affiliations:** Key Laboratory of Materials Physics, Institute of Solid State Physics, Chinese Academy of Sciences Hefei 230031 China wateratnanoscale@hotmail.com yyang@theory.issp.ac.cn; University of Science and Technology of China Hefei 230026 China

## Abstract

The structural, dynamical and electronic properties of water molecules on the β-PtO_2_(001) surface has been studied using first-principles calculations. For both water monomer and monolayer, the adsorption energies are found to be three to five times larger than that of water adsorption on the Pt surface, and the dissociative adsorption configurations are energetically more stable. The adsorption energies are positively correlated with the charge transfer between the water molecule and the substrate, and the charge-rebalance between the Pt and O atoms of β-PtO_2_ upon water adsorption. More interestingly, an exceptionally large redshift is observed in the OH stretching mode of the adsorbed water monomer, due to the very strong hydrogen bonding with the substrate. The strong water–substrate interactions have significant effects on the molecular orbitals of the chemisorbed water molecules.

## Abstract 2

The structural, dynamical and electronic properties of water molecules on the β-PtO_2_(001) surface has been studied using first-principles calculations.

## Graphical abstract

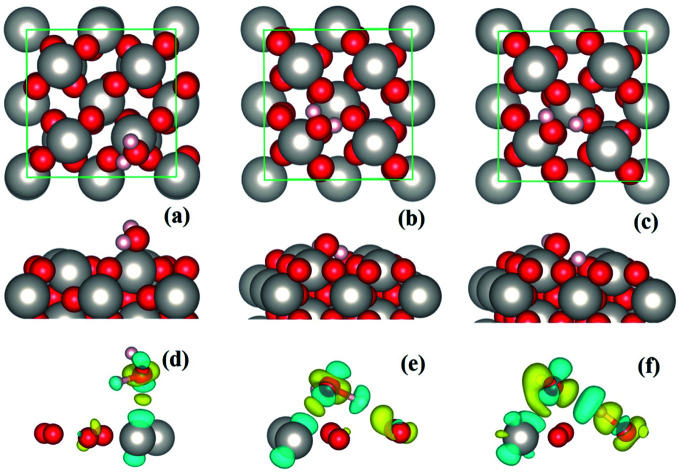

## 1. Introduction

The adsorption of water molecules on the surfaces of solid state materials is a ubiquitous phenomenon which plays an important role in modifying the surface structure and consequently the stability and reactivity of the surfaces. The presence of water molecules has significant influences on the interactions of surfaces with the other substances.^1,2^ As a product of the oxygen reduction reaction (ORR) that takes place in fuel cells, water naturally presents on the surfaces of the electrode. Platinum (Pt) is the most commonly employed material for the electrode, owing to its high reactivity for catalyzing the ORR.^3,4^ It has been found in previous works^5–8^ that the oxides of Pt can be formed on the Pt surface under oxygen rich conditions or at a potential-imposed interface such as the electrolyte–electrode interface of the proton-exchange membrane fuel cells after some time of application. Recent *in situ* and real-time experimental measurements^9^ have shown that thin films of Pt oxides which mainly consist of precursor of β-PtO_2_ are formed when the potential is ∼1.4 V with respect to the reversible hydrogen electrode (RHE).

Previous experimental and theoretical works have established that,^10,11^ water molecules are adsorbed molecularly (undissociated) as the wetting layer on Pt surface, with the adsorption energy of ∼0.30 eV for water monomer and ∼0.52 eV per H_2_O for water hexamer.^12^ To date, however, the adsorption structures of water on the surface of PtO_2_ remain unknown, which are practically more relevant to the ORR process on the electrode of fuel cell. In this work, we study the adsorption structures of sub-monolayer and monolayer water molecules on β-PtO_2_(001) surface. Our first-principles calculations show that, for both water monomer and monolayer adsorption, the molecular adsorption state is found to be energetically less stable than the dissociative adsorption state, in direct contrast to the molecular state of water adsorption on Pt electrode surface. Depending on the adsorption configurations, the adsorption energy of water molecules on PtO_2_(001) ranges from ∼1.0 eV to 1.7 eV, which is three to five times larger than the adsorption of water on Pt surface. The water–substrate interactions have significant effects on the vibrational frequencies and molecular orbitals of the adsorbed water molecules. In particular, an exceptionally large redshift in the frequency of the O–H stretching mode of water molecule, ν(OH), is observed in the adsorption configuration where a strong hydrogen bond is formed with the substrate. Quantitative analysis show that positive correlation exists between the binding strength of water on PtO_2_(001) and the charge transfer from water to the substrate.

The contents of this paper are organized as follows: after this brief introduction, the computational method employed in this study will be described in Sec. 2. The results and discussion regarding the adsorption structures of water molecules on β-PtO_2_ (001), the effects of water–substrate interactions on the vibrational properties and electronic structures of the adsorbed water molecules, will be presented in Sec. 3. The conclusion part is given in Sec. 4.

## 2. Computational methods

The crystal of β-PtO_2_ has a CaCl_2_-type structure, which can be synthesized under high pressure conditions.^13^ The experimentally determined lattice parameters are as follows:^13^*a* = 4.4839 Å, *b* = 4.5385 Å, *c* = 3.1360 Å, *α* = *β* = *γ* = 90° and *Z* = 2. The lattice parameters computed using density functional theory (DFT) are the following: *a* = 4.613 Å, *b* = 4.556 Å, *c* = 3.191 Å, *α* = *β* = *γ* = 90°. As show below, the effects of the small difference between experimental and theoretical lattice parameters are negligible. Therefore, unless specially stated, the experiment lattice parameters will be employed for the simulations. In our study, the β-PtO_2_(001) surface is modeled by separating the (2 × 2 × 3) supercell of β-PtO_2_ along the *c*-axis (*z*-direction) with a vacuum layer of ∼10 Å while the *a* & *b*-axis extend in the *xy*-plane using periodic boundary conditions. The atomic positions of the bottom three layers of the six-layer slab are fixed to simulate the bulk state. An isolated water molecule is simulated by placing it in a (10 Å × 10 Å × 10 Å) box. The first-principles calculations were carried out by the VASP code,^14,15^ which is based on DFT. A plane wave basis set and the projector-augmented-wave (PAW) potentials^16,17^ were employed to describe the electron wave function and the electron–ion interactions, respectively. The exchange–correlation interactions of electrons are described by the PBE type functional.^18^ The energy cutoff for plane waves is 600 eV. With reference to the results obtained using higher energy cutoffs (700 eV, 800 eV), the energy cutoff of 600 eV ensures the calculated adsorption energies to converge to within an error bar of ∼30 meV or better. For structural relaxation and total energy calculations of the H_2_O/PtO_2_ system, a 2 × 2 × 1 Monkhorst–Pack k-mesh^19^ is generated for sampling the Brillouin zone (BZ). An 8 × 8 × 8 k-mesh is employed for the calculation of an isolated water molecule.

The adsorption energy (*E*_ads_) of water molecules is calculated *via* the following formula:1
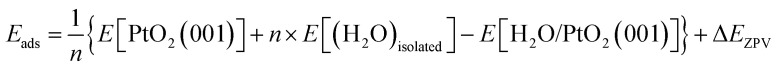
where *E*[H_2_O/PtO_2_(001)], *E*[PtO_2_(001)], *E*[(H_2_O)_isolated_] are respectively the total energies of the adsorption system, the PtO_2_(001) substrate, and an isolated water molecule; *n* is the number of water molecules contained in each simulation supercell. For the simulation of water monomer, *n* = 1; and *n* = 4 for the simulation of 1 monolayer (ML) adsorption, in which the surface unit cell contains four PtO_2_ units. The term Δ*E*_ZPV_ is the energy correction due to the change of zero-point energy of water molecules, from isolated state to adsorption state. In our calculation, Δ*E*_ZPV_ is computed as follows: 
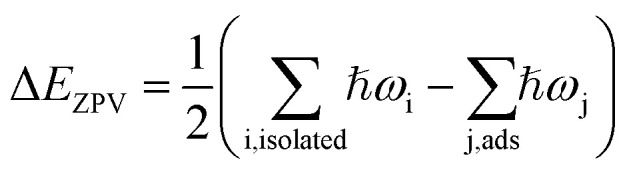
, where *ω*_i_ and *ω*_j_ are respectively vibrational frequencies of water molecules at isolated and adsorbed state, and *ℏ* is the Planck constant. The vibrational frequencies of the water molecules and the adsorption systems were computed using the density functional perturbation theory (DFPT).^20,21^

## 3. Results and discussion

**Fig. 1:**
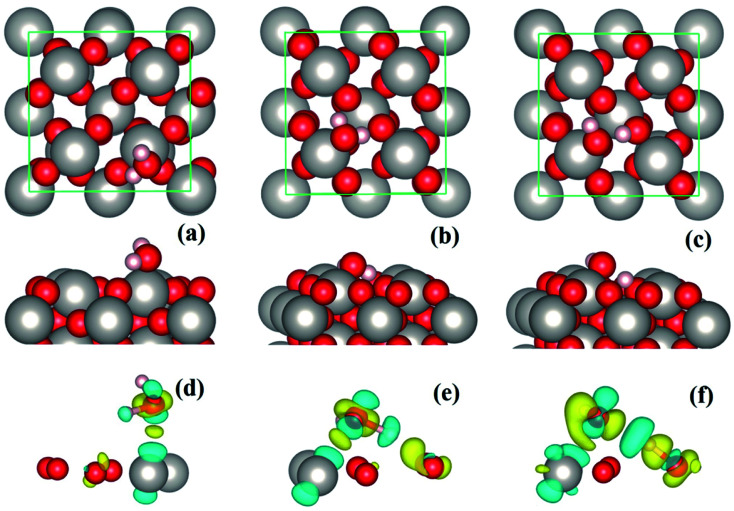
Panels (a–c): top view (upper panels) and side view (lower panels) of the molecular (a and b) and dissociative (c) adsorption of water monomers on β-PtO_2_(001). Right below the side view of each configuration, is the corresponding charge density difference (panels (d–f)). The Pt, O, and H atoms are represented by silver (largest), red (second largest), and white (smallest) spheres, respectively.

The PtO_2_(001) surface on which the water molecules are adsorbed (Fig. 1) has a rectangular surface unit cell, consisting of four line-shape O–Pt–O units which locate at the four vertices of the rectangle. Compared to the Pt atoms of bulk state which are coordinated by six O atoms, the Pt atoms on the surface are only coordinated by four O atoms. Such coordinatively unsaturated atoms serve as the adsorption sites of water molecules. For monomer adsorption, a number of configurations are considered, including the ones that stand upright on or parallel to the top sites of substrate atoms, forming O–Pt bonds and/or H–O hydrogen bonds. It turns out that the tilted lying configurations that form O–Pt bonds with the substrate are energetically favored over the upright standing ones. Three typical configurations, two molecular and one dissociative adsorption structures of water monomers, are schematically depicted in Fig. 1(a–c). The corresponding adsorption energies (*E*_ads_) and geometric parameters describing the adsorption configurations are tabulated in Table 1. The values of *E*_ads_ are ∼1 eV and above, which indicate that water molecules are chemically adsorbed on the PtO_2_(001) surface. The adsorption energies (∼1 eV to 1.7 eV) are three to five times that of water adsorption on Pt surface.^12^ In calculations using the theoretical lattice parameters of PtO_2_, the values of *E*_ads_ may differ by several tens of meV (*e.g.*, *E*_ads_ ∼ 0.99 eV for the configuration shown in Fig. 1(a), differs by ∼30 meV), which is negligible comparing to the magnitude of *E*_ads_. For an isolated or free state water molecule, the calculated HOH angle is ∼103.70° (experimental value:^1^ 104.52°) and the OH bond length is ∼0.972 Å (experimental value:^1^ 0.957 Å). Upon adsorption, the HOH angle is either enlarged (Fig. 1(a)) or contracted (Fig. 1(b) and (c)), and all the OH bond lengths are elongated. The molecular configuration (Fig. 1(b)) that forms a hydrogen bond with the substrate O (labeled as O^S^ hereafter) is much more stable than the one without hydrogen bonding (Fig. 1(a)). Given that the O–Pt bonds formed between water and the substrate are of similar strength, the strength of the hydrogen bond formed between water and the substrate can therefore be estimated *via* the difference of adsorption energy, which is ∼0.54 eV. To our knowledge, this is the strongest hydrogen bond ever found in OH⋯O systems. As shown in Fig. 1(c) and Table 1, the adsorption configuration shown in Fig. 1(b) is further stabilized by breaking the OH bond that forms hydrogen bond with the substrate and transferring the H atom to the underlying O^S^. As a result, a pair of hydroxyls (OH) which are connected by a hydrogen bond appear on the PtO_2_(001) surface.

**Table 1:** The adsorption energies and geometric parameters for the monomer and 1 monolayer (ML) water adsorption on β-PtO_2_ (001). For 1 ML adsorption, the averaged geometric parameters are listed with the standard derivation provided in the parentheses. ∠HOH: the HOH angle; OH^1^ and OH^2^: the length of the two OH bonds; ΔO_*xy*_: the lateral displacement of the adsorbed O away from the precise top of Pt; ΔO_*z*_: the vertical distance of the adsorbed O from the precise top of Pt; O^A^–Pt: the distance between the adsorbed O and Pt; H^A^–O^S^: the nearest distance between the H of adsorbed H_2_O and substrate O

Configuration	*E*_ads_ (eV per H_2_O)	∠HOH (°)	OH^1^ (Å)	OH^2^ (Å)	ΔO_*xy*_ (Å)	ΔO_*z*_ (Å)	O^A^–Pt (Å)	H^A^–O^S^ (Å)
Fig. 1(a)	1.02	106.32	0.996	0.978	0.928	1.955	2.164	1.915
Fig. 1(b)	1.56	97.18	1.058	0.996	1.417	1.533	2.088	1.512
Fig. 1(c)	1.71	99.60	1.718	0.977	1.226	1.575	1.996	1.017
Fig. 2(a)	1.56	97.14 (0.04)	1.058 (0.001)	0.993 (0.001)	1.411 (0.000)	1.534 (0.000)	2.084 (0.000)	1.519 (0.001)
Fig. 2(b)	1.66	96.99 (0.03)	1.673 (0.000)	0.981 (0.001)	1.262 (0.000)	1.544 (0.000)	1.995 (0.000)	1.024 (0.000)

To get insight into the water–substrate interactions, we have calculated the charge density difference for monomer adsorption, which is defined as follows:

2 Δ*ρ* = *ρ*[H_2_O/PtO_2_(001)] − *ρ*[PtO_2_(001)] − *ρ*[H_2_O],

where *ρ*[H_2_O/PtO_2_(001)], *ρ*[PtO_2_(001)] and *ρ*[H_2_O] are the electron densities of the H_2_O/PtO_2_(001) system, the PtO_2_(001) surface and the water molecule with the same geometry as the adsorption configuration, respectively. The spatial distribution of the charge density difference is shown in Fig. 1(d–f), for the three monomer adsorption configurations studied above. Clearly, stronger water–substrate (measured by *E*_ads_) interactions are accompanied by larger magnitude of charge redistribution. We tried to make quantitative analysis on the charge transfer between water molecule and the substrate, by performing Bader analysis^22,23^ on the charge densities of the adsorption systems, the PtO_2_(001) surface and the water molecule. The net charge transfer, Δ*q*(H_2_O), is defined as Δ*q*(H_2_O) = *q*(H_2_O)_ads_ − *q*(H_2_O)_isolated_, *i.e.*, the change in the total number of electrons after adsorption on PtO_2_(001). For the three adsorption configurations shown in Fig. 1(a–c), the value of Δ*q*(H_2_O) is calculated to be ∼−0.150*e*, −0.153*e*, and −0.571*e*, respectively. We have also analyzed the variation of the total number of electrons associated with the substrate O and Pt, and found that Δ*q*(O^S^) is ∼0.164*e*, 0.273*e*, and 0.761*e*, and Δ*q*(Pt) is ∼−0.016*e*, −0.121*e*, and −0.191*e* for the three configurations, respectively. It is clear that electrons are transferred from the water molecule (precisely, the H atoms of adsorbed water molecule) to the substrate O atoms. Moreover, the charges around the underlying Pt are also rebalanced by donating a small amount of electrons to the neighboring substrate O. Note that the small deviation from the charge-neutral condition (Δ*q*(H_2_O) + Δ*q*(O^S^) + Δ*q*(Pt) = 0) is simply due to numerical error. Nevertheless, the results demonstrate unambiguously that the water–substrate interactions are enhanced by charge transfer: larger *E*_ads_ is associated with larger Δ*q*(O^S^).

**Fig. 2:**
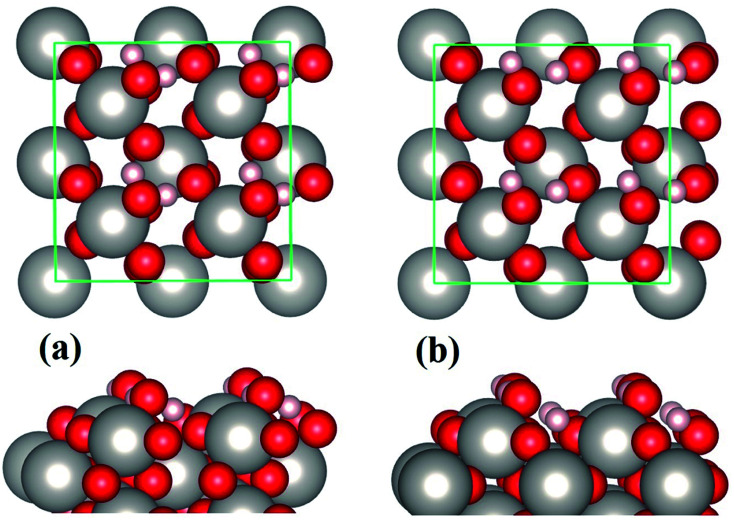
Molecular (a) and dissociative (b) adsorption of 1 ML water on β-PtO_2_(001).

In the case of water monolayer (ML) adsorption, two configurations are considered (Fig. 2): the molecular and dissociative type, in which the water molecules have similar geometries as the monomers shown in Fig. 1(b) and (c). As seen from Table 1, the 1 ML molecular adsorption has the same *E*_ads_ as the monomer configuration shown in Fig. 1(b), while the 1 ML dissociative adsorption has a value of *E*_ads_ slightly smaller (differs by ∼0.05 eV) than the dissociative monomer shown in Fig. 1(c). This is understandable when considering the following facts: the adsorption geometries of each water molecule of the 1 ML molecular configuration (Fig. 2(a)), such as the HOH angles, OH bond lengths, the positional displacement of the O atom in water molecule from the Pt top site, and the lengths of hydrogen bonds, are nearly identical to that of the monomer in Fig. 1(b); there are, however, minor differences between the geometric parameters of the 1 ML dissociative configuration (Fig. 2(b)) and that of the monomer dissociative adsorption in Fig. 1(c). On the other hand, for 1 ML adsorption, either molecular or dissociative, each constituent water molecule shares nearly identical adsorption geometries (Fig. 2 and Table 1). Due to the very strong water–substrate interactions (the order of 1 eV and above), the water–water interactions, *i.e.*, the hydrogen bonding between water molecules, consequently plays a minor role in determining the adsorption structures for monolayer and submonolayer coverages.

**Fig. 3:**
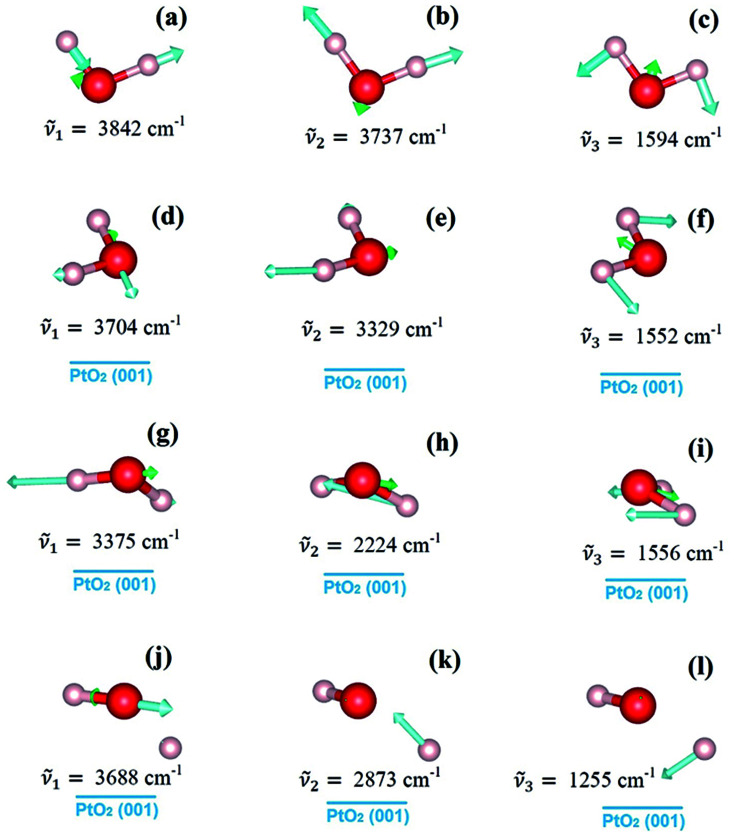
From top to bottom, the first three normal modes of vibration of an isolated water molecule (panels (a–c)), and the water monomers on β-PtO_2_(001) as shown in Fig. 1(a) (panels (d–f)), Fig. 1(b) (panels (g–i)), and Fig. 1(c) (panels (j–l)), respectively.

The strong water–substrate interactions have significant effects on the adsorption structures of water molecules and therefore the vibrational properties and electronic structures. Shown in Fig. 3, are the three normal modes of vibration of an isolated water molecule, together with the corresponding normal modes of vibration of the adsorbed water monomers on PtO_2_(001), in the order of vibrational frequencies. For an isolated water molecule, the asymmetric OH stretching mode (*ν*_a_(OH)) has the highest frequency (*ṽ*_1_ = 3842 cm^−1^), the symmetric OH stretching mode (*ν*_s_(OH)) is second highest (*ṽ*_2_ = 3737 cm^−1^), and the HOH bending mode (*δ*(HOH)) is the third (*ṽ*_3_ = 1594 cm^−1^); all of which compare well (within an error bar of ∼2%) with the data reported in literatures (*ṽ*_1_ = 3756 cm^−1^; *ṽ*_2_ = 3657 cm^−1^; *ṽ*_3_ = 1595 cm^−1^).^1,24^

For the molecular adsorption of water monomer, significant redshifts of vibrational frequencies are found in both the asymmetric and symmetric OH stretching mode, while only minor changes are observed in the HOH bending mode (Fig. 3(d–i)). Firstly, the magnitude of redshift in the OH stretching modes (asymmetric or symmetric) of the molecular configurations, Δ*ν*(OH), is found to increases with *E*_ads_, and the absolute value of charge transfer Δ*q*. Secondly, the order of Δ*ν*(OH), can also be measured by the OH bond lengths (Table 1): for the same mode, longer OH bond lengths implies smaller vibrational frequency, or larger redshift Δ*ν*(OH). In addition, smaller redshift is observed in the asymmetric OH stretching mode of the same monomer configuration, *i.e.*, Δ*ν*_a_(OH) < Δ*ν*_s_(OH). The HOH bending/scissoring motions are least affected by the adsorption geometry which is confined on the PtO_2_(001) plane. Therefore, much smaller redshift is found in the bending mode.

Returning to the redshift of the OH stretching modes, we find that an exceptionally large redshift presents in the symmetric stretching mode of the water monomer shown in Fig. 1(b), with a vibrational frequency of *ṽ*_2_ = 2224 cm^−1^, which corresponds to a redshift Δ*ν*_s_(OH) = 1513 cm^−1^. To the best of our knowledge, this is the largest redshift of OH stretching mode ever reported for the water-based systems, in which the redshift of *ν*(OH) due to hydrogen bonds usually ranges from tens to several hundred cm^−1^ and typically ∼500 cm^−1^.^25–27^ The giant redshift can be explained by the very strong hydrogen bond formed between the adsorbed water molecule and the substrate O. As discussed above, the strength of the hydrogen bond is estimated to be ∼0.54 eV, being probably the strongest in the OH⋯O systems. The length of the related OH bond is elongated to be ∼1.058 Å, increased by 0.086 Å with comparison to the isolated one. The remarkable weakening of OH bond leads to the giant redshift in the *ν*(OH) mode.

In the case of dissociative adsorption, a pair of OH groups presents (Fig. 1(c) and (f)): the dangling OH from the adsorbed water molecule, and the new OH group formed by the transferred H and substrate O^S^. By comparing the frequency of their OH stretching modes (Fig. 3(j) and (k)), *i.e.*, 3688 cm^−1^*versus* 2873 cm^−1^, a large redshift in *ν*(OH) is observed, which is Δ*ν*(OH) = 815 cm^−1^. Such a large redshift in the OH stretching is again due to the strong hydrogen bonding interactions between the dangling OH and the newly formed OH on the substrate, which can be schematically denoted as O⋯HO^S^. Another feature characterized by the vibration mode is the absence of HOH bending mode, which is typically ∼1500 cm^−1^ for the molecularly adsorbed states (Fig. 3(f) and (i)). Instead, one sees a vibration mode relating with the bending motion of the HO^S^ group on the PtO_2_(001) surface (Fig. 3(l)), with a frequency of *ṽ*_3_ = 1255 cm^−1^.

**Fig. 4:**
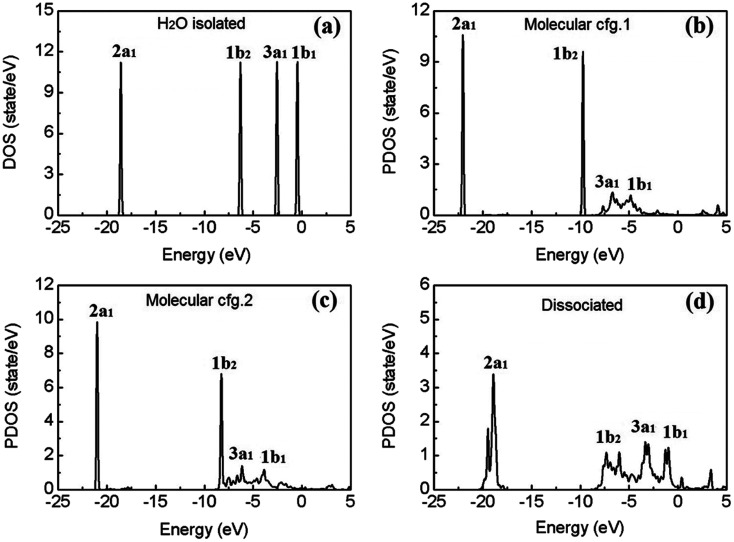
Calculated electron density of states (DOS) of an isolated water molecule (a), and the site-projected DOS (PDOS) of the molecular (b and c) and dissociated (d) water monomers on β-PtO_2_(001). The Fermi level of the system is set at 0 eV.

Fig. 4(a) shows the calculated electronic density of states (DOS) of an isolated water molecule. From left (deep level) to right (shallow level), the four discrete peaks/energy levels of valence electrons correspond to the so-called molecular orbitals (MOs) named as 2a_1_, 1b_2_, 3a_1_, and 1b_1_, respectively. In the picture of linear combination of atomic orbitals (LCAO), the 2a_1_ and 3a_1_ MOs mainly consist of the 1s orbitals of the two H atoms, the 2s and 2p orbitals of the O atom; the 1b_2_ MO comprises mainly of the 1s orbitals of H and the 2p orbitals of O; and finally 1b_1_, the highest occupied molecular orbital (HOMO), consists mainly of the 2p orbitals of O, which is usually called the “lone pair” of electrons.

To explore the effects of water–substrate interactions on the MOs, the site projected DOS (PDOS) of the water monomers on PtO_2_(001) are displayed in Fig. 4(b–d), for the molecular and dissociative configurations. Hereafter, the molecular adsorption configuration depicted in Fig. 1(a) is indicated as configuration 1 (abbr.: cfg.1), and that in Fig. 1(b) as configuration 2 (abbr.: cfg.2), for the simplicity of discussion. As a consequence of the strong water–substrate interactions, the energy levels near the Fermi level are broadened and the two MOs 3a_1_ and 1b_1_ overlaps with each other while the 2a_1_ and 1b_2_ orbitals remain untouched in molecular cfg.1. The deeper MO 1b_2_ overlaps slightly with 3a_1_ and 1b_1_ in molecular cfg.2, where the binding with substrate is stronger. In the case of dissociative adsorption where the strongest water–substrate interactions present, the three MOs 1b_2_, 3a_1_ and 1b_1_ are broadened and deeply mixed with each other (Fig. 4(d)). In addition, the deepest valence MO 2a_1_ is also perturbed and modified.

**Fig. 5:**
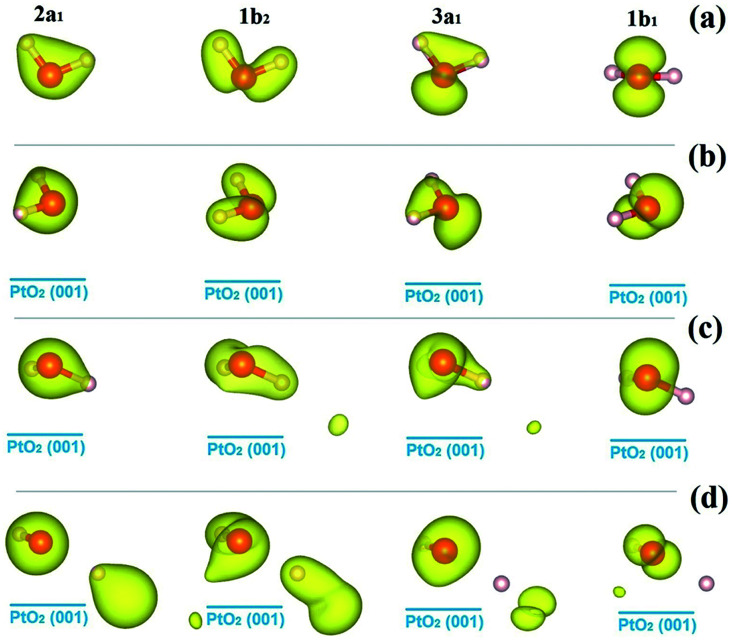
Isosurfaces of the charge densities of the molecular orbitals of water molecules, |*ψ*_MO_|^2^, as identified in Fig. 4, for the isolated state (a), and the molecular cfg.1 (b), cfg.2 (c), and dissociative (d) monomers on β-PtO_2_(001). The isovalue for charge density plotting is 0.035*e*/(Bohr)^3^.

We go further to study the influence of water–substrate interactions on the wave functions of the MOs (*ψ*_MO_), by investigating the spatial distribution of the charge densities, |*ψ*_MO_|^2^, as plotted in Fig. 5, for the isolated water molecule (Fig. 5(a)) and the adsorbed ones (Fig. 5(b–d)). The charge density of the MOs of an isolated water molecule is simply the partial charge density of each valence level as shown in Fig. 4(a). For the MOs of an adsorbed water monomer, the corresponding charge density is obtained by subtracting the partial charge density of the substrate PtO_2_ from the whole H_2_O/PtO_2_ system within a specified energy interval as indicated by the PDOS shown in Fig. 4(b–d). The *lm*-character (s, p, …) of the MOs can be obtained by projecting the wave functions onto the spherical harmonics. The results are displayed in Table 2, which describe the major characteristics of the wave functions contributed from the atomic orbitals. As expected, the MOs (3a_1_, 1b_1_) near the Fermi level are mostly affected and the 2a_1_ orbital which locates far away from the Fermi level is the least perturbed with comparison to the isolated molecule. Aside from the variations in the occupation numbers of the s and p_*x*_, p_*y*_, p_*z*_-orbitals, which are partly due to the rotation of coordination systems where the spherical harmonics are represented, only minor changes are found in the sum of the *lm*-components of the 1b_1_ orbitals of the molecularly adsorbed monomers.

**Table 2:** Calculated *lm*-components of the wave functions of the water molecular orbitals (MO) of the isolated state, molecular and dissociative adsorption on β-PtO_2_(001) surface. The abbreviation “cfg” represents “configuration”. The sum of the *lm*-components of each MO, *lm*-sum, is also presented

State of H_2_O molecule	Molecular orbitals	s	p_*y*_	p_*z*_	p_*x*_	*lm*-sum
Isolated	2a_1_	0.896	0.050	0.000	0.031	0.977
	1b_2_	0.331	0.066	0.000	0.516	0.913
	3a_1_	0.182	0.513	0.000	0.055	0.750
	1b_1_	0.000	0.000	0.691	0.000	0.691
Molecular cfg. 1	2a_1_	0.874	0.037	0.012	0.017	0.940
	1b_2_	0.298	0.049	0.198	0.313	0.858
	3a_1_	0.109	0.258	0.185	0.081	0.633
	1b_1_	0.050	0.297	0.204	0.193	0.744
Molecular cfg. 2	2a_1_	0.821	0.032	0.011	0.012	0.876
	1b_2_	0.239	0.177	0.024	0.274	0.714
	3a_1_	0.156	0.305	0.094	0.163	0.718
	1b_1_	0.037	0.091	0.488	0.151	0.767
Dissociative	2a_1_	0.912	0.033	0.005	0.013	0.963
	1b_2_	0.350	0.392	0.144	0.102	0.988
	3a_1_	0.059	0.083	0.273	0.301	0.716
	1b_1_	0.005	0.083	0.138	0.193	0.419

By contrast, one sees significant decrease in the sum of the *lm*-components of the 1b_1_ orbital of the dissociative water monomer. Such changes may be attributed to the mixing of the MOs, and the intrinsic incompleteness of the atomic orbitals in expanding the MOs' wave functions. Owing to the strong water–substrate interactions, the former MOs near the Fermi level are deeply mixed with each other and form new MOs, which we provisionally name as “3a_1_ + 1b_1_” for molecular cfg.1 and cfg.2, and “1b_2_ + 3a_1_ + 1b_1_” for the dissociated one. Within the new MO with modified PDOS, gapless transition between the neighboring energy levels can happen. From the data listed in Table 2, the sum of *lm*-components of the new MO 3a_1_ + 1b_1_ is 1.377 and 1.485 for molecular cfg.1 and cfg.2, and is 2.123 for the new MO 1b_2_ + 3a_1_ + 1b_1_. On average, the sum of *lm*-components for one orbital is 1.377/2, 1.485/2, and 2.123/3, ∼70% of the full occupation, which is 1 by considering the normalization of electron wave function. Similar situation is found in describing the HOMO of an isolated water molecule (Table 2). This originates from the fact that the sp-orbitals projected on the waver functions of the MOs are not a complete basis set and therefore inevitably miss some features. Based on the analysis above and the data in Table 2, one can find that the energy center of HOMO is pushed down due to the mixing of the MOs near Fermi level and the formation of new MOs. From the point of energy, such down-shift of the occupied energy levels of electrons helps further stabilize the adsorption configuration.

## 4. Conclusion

To summarize, we study the adsorption of water molecules on β-PtO_2_(001) surface using DFT calculations. It is found that both monomer and monolayer water molecules are chemically adsorbed, with much larger adsorption energies than the adsorption on Pt, and the dissociative configurations being energetically favored, which is different from the case of water on Pt surface. Detailed analysis reveals that, the strength of water–substrate interactions are positively correlated with the magnitude of water–substrate charge transfer, and the charge-rebalance between the substrate Pt and O atoms. Due to the very strong hydrogen bonding interactions between the molecularly adsorbed water monomer and the PtO_2_(001), a giant redshift in the vibrational frequency of the OH stretching mode is observed. The strong water–substrate interactions also have significant effects on the electronic structures of the adsorbed molecules, in which the energy levels near the Fermi level are broadened and the molecular orbitals are deeply mixed. We expect that results presented here can be tested by traditional vibrational spectroscopies (*e.g.*, Raman and infrared),^1^ ultraviolet photoemission spectroscopy measurements, as well as the recently developed scanning tunneling microscopy which is capable of imaging the molecular orbitals of water with subatomic resolution.^28^ The adsorption of multilayer water molecules on the PtO_2_(001) surface, and their effects on the ORR in fuel cell applications will be the subject of future research.

## Conflicts of interest

The author declares no conflicts of interest.

## Supplementary Material
